# Atmospheric Deposition-Carried Zn and Cd from a Zinc Smelter and Their Effects on Soil Microflora as Revealed by 16S rDNA

**DOI:** 10.1038/srep39148

**Published:** 2016-12-13

**Authors:** Feng Shen, Yanxia Li, Min Zhang, Mukesh Kumar Awasthi, Amjad Ali, Ronghua Li, Quan Wang, Zengqiang Zhang

**Affiliations:** 1 College of Natural Resources and Environment, Northwest A&F University, Yangling, Shaanxi, China; 2 School of Environment, Beijing Normal University, Beijing, China

## Abstract

In this study, we investigated the influence of heavy metals (HM) on total soil bacterial population and its diversity pattern from 10 km distance of a Zinc smelter in Feng County, Qinling Mountain, China. We characterized and identified the bacterial community in a HM polluted soil using 16S rDNA technology. Out results indicated that the maximum soil HM concentration and the minimum bacterial population were observed in S2 soil, whereas bacterial diversity raised with the sampling distance increased. The bacterial communities were dominated by the phyla *Proteobacteria, Acidobacteria* and *Actinobacteria* in cornfield soils, except *Fimicutes* phylum which dominated in hilly area soil. The soil CEC, humic acid (HA)/fulvic acid (FA) and microbial OTUs increased with the sampling distance increased. *Shewanella, Halomonas* and *Escherichia* genera were highly tolerant to HM stress in both cultivated and non-cultivated soil. Finally, we found a consistent correlation of bacterial diversity with total HM and SOM along the sampling distance surrounding the zinc smelter, which could provide a new insight into the bacterial community-assisted and phytoremediation of HM contaminated soils.

Eco-environmental pollution caused by smelting, mining and associated anthropogenic activity is a worldwide problem and scientists have focused more on particular environmental problems caused on the soil surface environment by the release of harmful substances, especially heavy metals (HM)^1^,^2^. In addition, HM deposition caused soil erosion and the destruction of vegetation on cultivated land by mining and smelting and as result HM accumulation in the food chain continues, which also poses a serious health hazard. Currently, great concern has been growing about waste dispersion and soil contamination from the smelting industry, particularly in relation to determining whether HM are deleterious to soil microbial diversity, and if so, to which microbes and how many. HM leach from the contaminated upper soil, percolate from the soil to the plants, and accumulate in the surrounding plant rhizosphere^3^. A previous study has reported that HM ions are immobilized in the soil by using various additives and plants is one of the better approaches to investigate soil HM contamination^4^,^5^,^6^. The accumulation of highly toxic HM in soil affects the microbial diversity, the soil nutrient supply and food production^7^,^8^,^9^. Additionally, the bioavailability of each HM also affects the soil microbial diversity^10^. Some studies have already noted that soil microorganisms can be a good indicator of soil quality and microbial diversity has been analyzed using either traditional culture methods and community-level physiological profiling or sole carbon source tests (e.g., Biolog)^11^,^12^.

However, all of these traditional methods may provide an inaccurate soil microbial profile because many species might be not detected. Some researchers have used biomass and changes in respiration as indicators of soil pollution and microbial diversity^13^,^14^, but this does not provide an exact assessment of the changes in diversity and functionality of the microbial biomass and may not be a useful indicator of the effects of HM contamination. On the other hand, SOM can also have great impact on microbial activity under HM-polluted conditions because SOM can bind with HM or toxic elements and alleviate toxicity as well as change their behavior in the soil^15^. In last few years, it became evident that bacterial diversity in the soil could be directly influenced by HM mobility and bioavailability by the production of bio-chelators such as bio-surfactants^16^.

Fulvic acids (FA), humic acid (HA), humin carbon (HC) and some insoluble components are also an important part of the SOM and significantly affect the HM binding capacity^17^. In addition, both saprophytic^18^,^19^ and mycorrhizal fungi^20^,^21^ have been shown to have differential responses to HM, including Pb, Cu, Cd and Zn. However, many bacterial species have proven tolerant to Zn while some are sensitive to Cu^20^,^22^,^23^, which is often an active ingredient in smelter waste. However, several earlier studies have explored the mobility of inorganic pollutants or HM in smelter site ground material by sequential extraction^24^. Similarly, studies that targeted arbuscular mycorrhizal fungi (AMF) variability in HM mines in a lead and zinc region have reported *Sophora viciifolia* rhizospheric microbiota interactions with the HM contaminated soil^21^ and the role of glomalin-related soil protein (GRSP) in HM sequestration^15^.

Nevertheless, these experiments provided indirect information on the carrier phases and influence of HM on the overall microbial diversity and the structure of the soil bacterial community. However, limited information was available on the role of SOM fractions such as HA, FA on HM availability and its consequences regarding soil bacterial diversity.

Previous research^25^,^26^ has indicated that the distance gradient is more greatly affected by HM deposition than wind direction, so we hypothesized that SOM fractions (HA, FA and HC) and the atmospheric deposition of HM (Cu, Cd, Ni, Pb and Zn) in the soil would cause abnormalities in the soil bacteria diversity. Therefore, our primary objectives were: (i) To investigate the effects of the HA, FA, HC and HM concentrations on soil bacterial diversity along a distance gradient from a zinc smelting area, and (ii) to compare the possible effects of HM accumulation on the entire soil bacterial community with the area adjacent to the zinc smelters using 16S rDNA technology. Our study also encompassed the mobility of pollutants and biogeochemical parameters.

## Materials and Methods

### Site description and soil sampling

**Figure 1:**
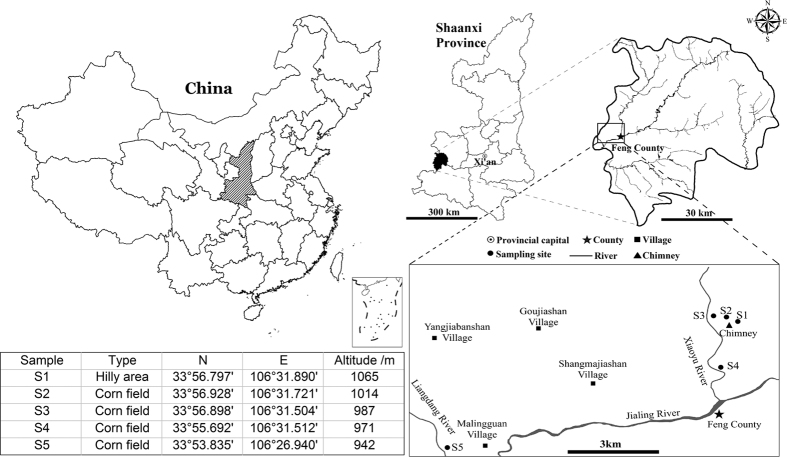
Sampling sites in the Dongling zinc smelter area. The smelter is located in Feng County, Qinling Mountain area of Shaanxi Province of China. The distance from the main chimney of S1 to S5 was 0.05, 0.05, 0.50, 2.00 and 10.00 km respectively. The maps within Fig. 1 were created using “ESRI’s ArcGIS 9.0 software (http://www.esri.com/software/arcgis)”.

In this study, five composite soil samples were collected at five distances from a typical zinc smelter located in the Feng County in Shaanxi Province, China to achieve a pollution gradient. Feng County is located in the central fold system of Qinling Mountain, which is the fourth largest zinc production area and the geographical boundary between northern and southern China. It is a hilly area, and the Jialing River passes across the border from the Northeast to Southwest in the same direction as the change in the slope. The climate of the region is a mountain climate with a subtropical humid monsoon climate as the basis, with obvious changes with elevation, and four distinct seasons. The annual average temperature is 11.4 °C (−1.1 °C in winter and 22.7 °C in summer) and the average rainfall is 613.2 mm, with a frost-free period of 188 days. The prevailing wind direction in Feng County was east and the wind speed varied from 3.4 to 7.6 ms^−1^ during the sampling period. This smelter has a history of over 20 years and an annual output of 150000 tons of zinc ingots and 1 million tons of coke and is still in operation. The forest coverage rate of Feng County is 75.8% with more than 360 types of trees and shrubs. Each sampling site was in a cornfield after the harvest, except that the first sampling site was in a hilly area. Five soil samples were taken at gradually increasing distances of 0.05, 0.05, 0.50, 2.00, and 10.00 km from the smelter and were designated as S1, S2, S3, S4 and S5 as shown in (Fig. 1). The S5 site was 10 km from the zinc smelter and was considered as the control. Five random soil subsamples were taken from each site from the top 20 cm using a stainless steel spatula and immediately mixed thoroughly. Then, the soil samples were taken back to laboratory for further analysis. An aliquot of the soil sample was stored at 4 °C for the analysis of physicochemical properties, while another aliquot was preserved at −80 °C prior to DNA extraction.

### Physiochemical analysis

The pH and the electrical conductivity (EC) of an aqueous extract [1:2 (w/v) soil: water ratio] of the soil samples were analyzed using a pH meter with a glass electrode (INESA PHSJ-3F, China) and a conductivity electrode (INESA DDS-307, Shanghai, China). The soil texture was determined using a Malvern Mastersizer 2000 particle size analyzer to measure the particle size distribution with alcohol as dispersant. The soil organic matter (SOM) content was titrimetrically determined using a potassium dichromate external heating method^27^. Total Kjeldahl nitrogen (TKN), total phosphorus (TP) and total potassium (TK) were measured after H_2_SO_4_-H_2_O_2_ digestion via Kjeldahl distillation (KETUO KDY-9820, China) followed by titration, while TP was determined with a UV-vis spectrophotometer (Shimadzu UVmini-1240, Japan) and TK with a Flame photometer (Shanghai JK FP640, China). The cation exchange capacity (CEC) was measured using the method developed by Stewart^28^. The basic properties of the soil are listed in Table 1. The extractable humus fractions (HA, and FA) were analyzed per a modification of the method of Donisa, *et al*.^29^, and the residual was considered as HC. The carbon concentrations (HA, FA and HC) of all of the fractions and the SOM were measured using the K_2_Cr_2_O_7_-heating method described by Bao^27^. The total HM content in soil samples was determined with a tri-acid digestion method (HNO_3_, HCl, HClO_4_) at a volume ratio of 1:3:1 according to the USEPA Method 3051 A^30^ with minor modifications, and the digested samples were analyzed using an atomic absorption spectrophotometer (AAS; PERSEE TAS-990, China). The BCR sequential extraction procedure was used to extract the different species of HM per the modified method of Rauret^24^. The HM in the extractant in each step obtained from the BCR procedure were also analyzed using AAS.

### DNA extraction and purification

Intracellular DNA was isolated from collected soil sample according to^31^, with some modification. Five grams of fresh soil sample were mixed with 13.5 mL of autoclaved extraction buffer [pH 8.0, 100 mM Tris-HCl, 100 mM di-sodium EDTA, 100 mM sodium phosphate, 1.5 M NaCl, 1% hex-adecylmethyl ammonium bromide (CTAB)], 50 microliters (μL) of lysozyme of (100 mg·mL^−1^). After the addition of 0.1 mL Proteinase K (1 mg·mL^−1^) and 0.1 mL Sodium lauryl sulfonate (SDS) (2% v-v) the mixture was incubated at 37 °C for 30 min while being shaken horizontally at 150 rpm. While final concentration of and each tube was incubated at 65 °C in a water bath for 2 h with gentle end-over-end mixing every 15 min. The mixtures were then centrifuged at 2500 × *g* for 5 min and the supernatant was collected into a fresh tube and the debris was extracted twice or more and the supernatants were combined. The proteins were denatured by the addition of chloroform-isoamyl alcohol and DNA was purified three times with the mixture of phenol, chloroform and isoamyl alcohol^32^. It was achieved by shaking the sample until the emulsion was not formed and then subsequently centrifuge for 5 min at 2500 × *g*, and washed twice with 5 mL of cold 70% ethanol, dissolved in 100 μL sterile, deionized water and stored at 4 °C. To reduce the likelihood of chimera formation and interphase containing impurities, the isolated DNA was then size fractionated by agarose gel electrophoresis and DNA P 20 kb recovered using a Geneclean UNIQ-10 Spin kit (Sangon biological company, Shanghai, China) according to the manufacturer’s instructions.

### PCR amplification and sequence processing

Total bacterial 16S rDNA gene was amplified using the bacterial universal primers 515f forward (5′-GTGCCAGCMGCCGCGGTAAT-3′) and barcoded-806r reverse (5′-GGACTACHVGGGTWTCTAA-3′), which target a broad diversity of bacteria with few biases against particular groups according to a standard protocol^33^. The PCR mixture (final volume 50 μL) contained 20 μL Premix Ex Taq (Takara Biotechnology), 0.4 μL of each primer (10 μM), 4 μL of five-fold diluted template DNA (1–10 ng) and 25.2 μL-sterilized water. Polymerase chain reaction (PCR) was performed with an initial 1 cycle of denaturation (3 min at 94 °C), six touchdown cycles of 45 s at 94 °C, 60 s from 65 °C to 58 °C, 70 s at 72 °C, followed by 22 cycles of 45 s at 94 °C, 60 s at 58 °C, 60 s at 72 °C with a final elongation of 72 °C for 10 min. Finally, the PCR products were purified by Wizard SV Gel and PCR Clean-up system (Promega, San Luis Obispo, CA, USA). The concentrations of the PCR products were fluorometrically quantified by the Qubit dsDNA HS Assay Kit (Invitrogen, Carlsbad, CA, USA) before being sequenced on the Miseq platform (Illumina, San Diego, CA, USA), at Novogene, Beijing, China. Raw sequences were processed in QIIME 1.7.0^34^. Sequences were quality trimmed and clustered into operational taxonomic units (OTUs) at a 97% identity threshold using UCLUST^35^. Representative sequences from individual OTUs were then aligned against the Green-genes core set^36^ using PyNAST^37^. Taxonomic assignment was carried out with the RDP Classifier^38^. The principal coordinate analysis (PCoA) was used to visualize the Bray-Curtis dissimilarity matrices based on the 97% OTU level across different copper concentrations^34^. Diversity was characterized by calculating richness (OTU numbers, Shannon index) and evenness (Gini coefficient). The Gini coefficient (ranging from 0 to 1) is a value to assess the specific degree of evenness, and a higher Gini coefficient indicates lower evenness of a community^39^. The Gini coefficient was calculated by performing ineq package in R3.0.2 software (http://www.r-project.org/).

### Data analysis

All statistical analyses were performed on triplicate and are expressed as the mean with the standard error of mean. The chemical and microbiological data were analyzed using an ANOVA with treatment as the independent variable. Significant differences of all variables between the different treatments were established by Tukey’s test when the variance was homogeneous and by Games-Howell’s test if the variance was not homogeneous. The correlation matrix for all chemical and biochemical parameters was calculated and the significance level was reported (*P* ≥ 0.01 and *P* ≥ 0.05) based on Pearson’s coefficients. The ARDRA patterns were analyzed with the Gel Compare II V.3.00 (Applied Math) software program and all gels were normalized prior to analysis. The genetic similarity of the bacterial populations in each sample was determined by comparison of the presence and absence of bands. Dice’s coefficient was used to calculate the resemblance matrix, while the un-weighted pair-group method using arithmetic means (UPGMA) was selected as the fusion strategy for elaborating the dendrograms.

## Results and Discussion

### Soil physicochemical properties

**Figure 2:**
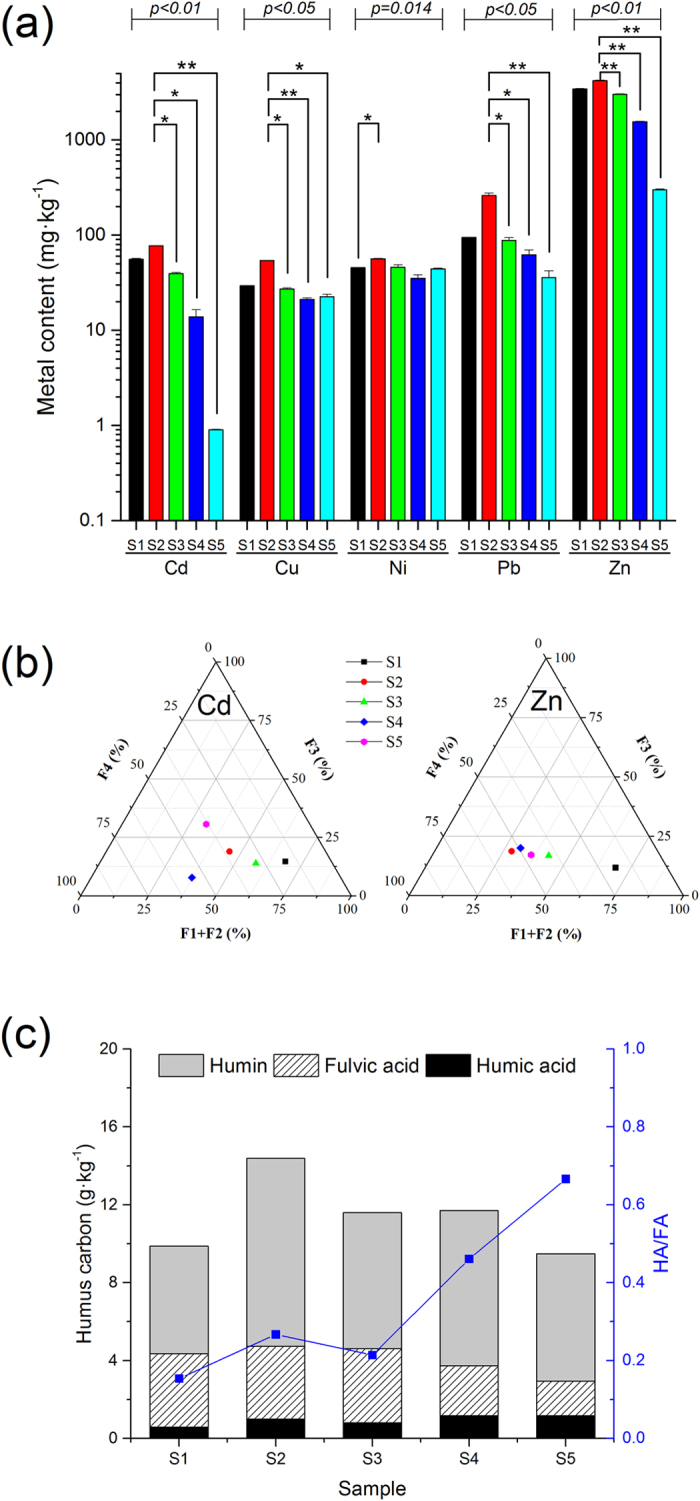
Total heavy metals, available heavy metals (Cd and Zn) and soil organic fractions in all five soil samples of zinc smelter. (**a**) Total heavy metals and grouped column chart with Y axis expressed as log 10 illustrates the heavy metals contents data collected for five different samples. This graph shows use of the “asterisk bracket” object for indicating significant differences between samples. (**b**) Triangular diagrams showing heavy metal (Cd and Zn) speciation in different soils. F1-acid soluble, F2-reducible, F3-oxidizable, F4-residual. (**c**) Humus carbon composition, humic acid and fulvic acid index in different soil samples.

Five different soil samples were collected from the Zn smelter polluted site and the physicochemical characteristics are listed in Table 1. The bacterial diversity of the HM contaminated soil was highly variable because it depended on the soil chemistry and environmental factors such as pH and moisture, which indicted a direct correlation between the soil properties and geographic variables and the soil bacterial diversity and community. Meanwhile the variation in the soil texture and physicochemical properties was consistently associated with the soil bacterial diversity and taxa with sampling distance (Fig. 2a and Table 1), This result was quite similar to the results of Drenovsky, *et al*.^40^ and Lauber, *et al*.^41^.

**Figure 3:**
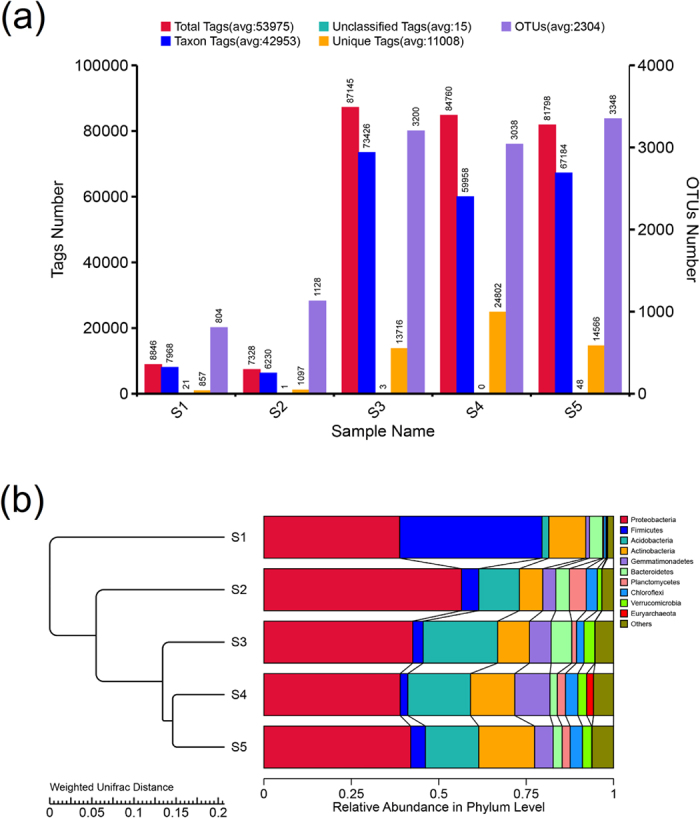
Basic information of the microbial communities in soils. (**a**) Tags and OTUs number statistics of different samples. all OTUs with an average relative read abundance >1% and total tags number (red) refers to the filtered the splicing sequence number. Taxon Tags (blue) refers to the number of Tags for building OTUs and access to classified information. Unclassified Tags (green tea) for building number of Tags OTUs but without access to classified information; Unique Tags (orange) refers to the frequency is 1, and can’t be clustering to the number of Tags OTUs; All the above corresponding with the vertical axis on the left, meanwhile the right vertical axis is the Number of OTUs (purple) refers to the Number of OTUs finally got. (**b**) The relative abundance of the dominant bacterial taxonimic groups seprated using total 16S rDNA gene sequences. Bacterial phyla level (top 10) and clustering tree based on the Weighted Unifrac distance assesed based on their relative abudance across all sampels and dominant groups were chosen for each having grater relative abundance. Relative abundance were estimted based on frequecny of occurrence of sequences classified to each taxonomic group.

The soil collected from S1, S2 and S3 appeared to have a silty texture, while the soil from S4 and S5 had a silt loam texture according to the United State system Malvern Mastersizer 2000 particle size analyzer. The pH of all of the soil samples was slightly alkaline, ranging from 8.45 to 8.68, and the EC was approximately similar, as shown in Table 1, while the CEC showed major differences between S1(16.3 cmol·kg^−1^) and S5(34.3 cmol·kg^−1^) and S2(25.5 cmol·kg^−1^), S3(25.9 cmol·kg^−1^) and S4(28.3 cmol·kg^−1^). The SOM content also decreased with distance and the lowest SOM content was found at S5(16.3 g·kg^−1^), 10 km from the zinc smelter. The soil from S2 (0.05 km from the smelter) had the highest SOM content (24.8 g·kg^−1^) followed by S3(20.2 g·kg^−1^), S3(20.0 g·kg^−1^) and S5(16.3 g·kg^−1^). However, the soil physicochemical properties of all five samples did not show major variations, but the relative abundance of bacterial diversity and taxa showed significant variations with an increase in the sampling distance. Nevertheless, differences in bacterial diversity may occur because of the soil texture and SOM because the clay and silt content combined with a high HM content and SOM affect soil textural characteristics such as porosity and stability^9^,^42^,^43^. Hassan^44^ and Liao^45^ reported that the soil texture directly affects soil physical properties such as temperature, gas exchange and the soil water holding capacity, which consequently affects the soil nutrient availability and microbial diversity. Boivin^46^ reported that natural soil properties generally confounded the response of bacterial communities to metals in a field situation. The results of our study also suggested that the soil texture and the SOM content played an important role in the variation of bacterial diversity in all five soil samples, as shown in Table 1 and Fig. 3a,b. However, a previous study^9^ reported that the SOM fraction (HM, HA and FA) showed major differences in the taxa, OTU_S_ and soil bacterial diversity rather than for just a few taxa, with increasing distance from the zinc smelter. Consequently, He, *et al*.^47^ also reported that the SOM concentration significantly affected HM availability and altered the bacterial abundance surrounding the smelter as a result.

The maximum TKN content was observed in S3 followed by S2, S5, and S4, while the lowest TKN content was found in S1. However, S3 also had the highest value for TP and TK (0.703 g·kg^−1^ DW TP), S1 and S5 were in the same range (0.605 g·kg^−1^ DW TP and 0.607 g·kg^−1^ DW TP, respectively), while S2 and S4 were also approximately in the same range (0.575 g·kg^−1^ DW TP and 0.576 g·kg^−1^ DW TP). A similar trend was also observed for TK, but S1 had the maximum value (6.025 g·kg^−1^ DW) and S5 had the lowest TK content (4.164 g·kg^−1^ DW TK). S3 was slightly lower than S1 (5.644 g·kg^−1^ DW TK), while S2 and S4 soil were close to each other (5.380 g·kg^−1^ DW TK and 4.944 g·kg^−1^ DW TK, respectively). Significant correlations were found among different parameters and a positive correlation was observed between pH and EC (*r* = 0.10, *P* = 0.034), TKN and TP (*r* = 0.98, *P* = 0.030), TKN and TK (*r* = 0.95, *P* = 0.012), and an inverse correlation between EC and CEC (*r* = −0.94, *P* = 0.020). Drenovsky, *et al*.^40^ and Cookson, *et al*.^48^ reported that the contents of soil nutrients play crucial role in soil bacterial diversity because the TKN, TP, TK content of each soil sample were directly correlated with essential and variable requirements of individual taxa and phyla. Several previous researchers have reported that each bacterial phylum or species had a different nutrient requirement for its metabolic activity, and as result, the optimum nutrient content is variable for each species and bacterial abundance also varies accordingly^47^. Despite a marginal difference in the chemical properties of each soil, the chemical properties strongly affected bacterial diversity in the soil around the smelter. In addition to the evidence in this study, several earlier researchers have reported that the soil nutrient concentration significantly affected the bacterial community diversity as evidenced by changes in the relative abundance of certain taxa^42^,^49^.

The total HM content of S2 was higher than S1, which was same distance to the main chimney as S2, as shown in Fig. 2a, then the HM content varied in the order S3 > S4 > S5 with distance. The HM content at S5, which was situated in a valley adjacent to Tujiaya 10 km from the smelter, was within the standard for uncontaminated soil. In fact, from the aspect of the individual elements, Cd, Pb and Zn were more alike than Cu and Ni, as seen in Fig. 2a. The similarity of Cd, Pb and Zn was consistent with our observation that pollution with Cd, Pb or Zn are associated with each other, might be due to the associated minerals. Based on the environmental quality standard for soil (GB 15618-1995), which is a mandatory standard of the Chinese EPA, Cd and Zn pollution is considered considerably more serious than pollution with other metals. The maximum levels of Cd, Cu Ni, Pb and Zn were 1.0, 400, 200, 500 and 500 mg·kg^−1^ respectively according to the standard^50^, and the total metal contents in S2 were 77.55, 0.14, 0.28, 0.52 and 8.44 times above the standard, respectively. Therefore, we chose Cd and Zn to show morphological analysis using the BCR method.

In this study, we divided the HM speciation into acid soluble, reducible, oxidizable and residual fractions based on the concentrations of the metals extracted using the BCR technique. The first two acid soluble fractions consisted of adsorbed ions on the ion exchangeable phases, while those associated with carbonate minerals and poorly crystalline minerals were considered to be the fractions readily bioavailable to microorganisms. The residual fraction of the HM is thought to be associated with stable minerals such as silicates and crystallized oxides of magnesium and aluminum and has the lowest mobility. Figure 2b is a triangle phase diagram that shows the distribution of Cd and Zn in the different fractions. In the diagram, the metal concentrations in the acid soluble (F1) and reducible fractions (F2) were combined in one point of the triangle because the metal in both fractions is likely highly mobile with changes in environmental conditions such as pH and EC. The other two points are the oxidizable (F3) and residual fractions (F4). All of the samples had similar distribution patterns of HM concentrations along the gradient for the absolute content of the F1 and F2 fractions, which indicates that Cd and Zn have a similar species distribution in different soils in all five samples, while the F3 fraction was less than 25%, and the F4 fraction was nearly less than 50%. The stable fraction (F4) was low and the acid soluble (F1) and reducible state fractions (F2) were high, suggesting that Cd and Zn have readily bioavailable fractions. The stable fraction (F4) was low and the acid soluble (F1) and reducible state fractions (F2) were high in the soil samples, especially in S1, suggesting that S1 had a high migration potential or bioavailability, followed by S3, S5 and S4. S2 was an exception; its bioavailability ranked in the middle for Cd and last for Zn, possibly due to increased fertilization to balance the reduction of crop yield caused by HM pollution, which altered the physicochemical properties.

The changes in the HC, HA and FA contents and the proportion of available metal fractions was determined for the five samples, and the results are shown in Fig. 2c. The concentration of HC gradually decreased from S3 to S5 with increasing distance from the main smelter chimney and the maximum HC content was observed in S2, while the lowest HC content was in S1. The HA content of the soil significantly increased with sampling distance. The maximum HA and FA were observed in S5 followed by S4 > S2 > S3 and were lowest in S1, possibly because of higher HM contamination because the sampling point was very close to the chimney. The FA content gradually decreased with distance and a relatively similar FA content was observed in S1, S2 and S3 and was lowest in S5. The similarity in the first three samples and the lower FA content in S4, might be due to difference in the deposition of HM with distance. The HAs displayed bidentate bridging or even bidentate chelation in low oxidation degree, but had a high oxygen functional group content in the high oxidation degree^51^. Compared to the HC and FA content, the HA content in S2 was significantly lower, but the HA content increased with distance. Lin^52^ reported that HA/FA had significant effects on metal ion adsorption by carbon nanotubes (CNTs) because aromatic monomers and the hydrophobic fraction of humic substances have a strong affinity for CNTs because of hydrophobic and π–π interactions, and the hydrophilic fractions of HA/FA contain functional groups such as carboxyl, phenol, hydroxyl, amine and quinine groups that can bind HM. Despite the differences, the common functional groups such as carboxyl, phenol, hydroxyl, amine and quinine groups make it possible that HA and FA have a strong complexation ability for HM that decreases their bioavailability and affects bacterial diversity.

### Composition of bacterial communities

The total bacteria community composition in the soil samples was compared among the five different sampling sites at increasing distances from the zinc smelter, and the results of a two-way ANOVA are shown in Fig. 3a. Significant differences in the bacterial community composition were observed among all five sampling sites (*p* < 0.01). Subsequently, comparisons were made between the bacterial communities in the soil samples based on the presence or absence of individual total Tags (avg: 53975), Taxa Tags (avg: 42953), Unclassified Tags (avg: 15), Unique Tags (avg: 11008) and OTUs (avg: 2304), and the results are shown in Fig. 3a. Figure 3 clearly indicates that the maximum total Tags (avg: 87145) were in S3, while S4 (avg: 84760) and S5 (avg: 81798) were slightly lower than the S3, but S1 and S2 were significantly lower than the other samples. Maximum taxa tags were found in S3, followed by S5, S4 and S1, with the least in S2. The highest number of Unique tags was observed in S4 followed by S5 and S3, but a very limited number of Unique tags were in S1 and S2.

The maximum 3348 and 3200 OTUs were in S5 and S3, while the number of OTUs significantly decreased with the distance from the zinc smelter, except that S4 also had a high value of 3038 OTUs. S5 and S3 accounted for most of the OTUs (48.5%) and an another relatively large proportion of OTUs (18.6%) was observed in S4. Remarkably, all OTUs with an average relative read abundance >1% and total number of tags (red) refer to the filtered splicing sequence number. Taxa Tags (blue) refers to the number of Tags for building OTUs and access to classified information. Unclassified Tags (green) refers to the number of OTU tags but without access to classified information; Unique Tags (orange) refers to a frequency of 1, and cannot be clustered to the number of OTU tags. The various Tags are on the vertical axis on the left, and the right vertical axis is the Number of OTUs (purple), which refers to the final Number of OTUs. The four most abundant bacterial groups found in all five of the samples were *Proteobacteria, Acidobacteria, Actinobacteria* and *Firmicutes*, which correspond to the top four reported by a previous study^21^ in zinc mines near the Feng County sampling site. However, some previous studies^21^,^53^,^54^ reported that the bacterial OTUs and taxa in surrounding the zinc smelter and the HM-contaminated soil significantly decreased with a higher concentration of HM. In addition, the results of Zheng, *et al*.^55^ indicated that HM pollution could increase the number of some indigenous bacterial OTUs and taxa when the concentrations of the SOM fraction*s* was higher in the soil. The bacterial community in all five of the soil samples was dominated by *Proteobacteria*, while *Acidobacteria* and *Actinobacteria* were predominant among the other phyla with increasing distance and HM contamination^56^,^57^.

The bacterial communities in all five soils samples had quite a similar diversity pattern, but different relative abundances were observed among the samples, as shown in Fig. 3b. It is clear that 9 dominant bacterial phyla (relative abundance >1%) were observed, including *Proteobacteria, Acidobacteria, Actinobacteria, Firmicutes, Gemmatimonadetes, Bacteroidetes, Chloroflexi, Planctomycetes*, and *Verrucomicrobia*, in all five samples. The results also indicated that the relative abundance of *Proteobacteria, Acidobacteria* and *Actinobacteria* was the highest, while the relative abundance of *Firmicutes* in S1 was higher than the group of bacteria above. All these nine phyla combined accounted for 95.11% of the sequences, while proportion of other phyla varied from 1.93% to 7.68% in the five samples. The relative abundance of the phyla was variable with distance. The percentage of *Proteobacteria* in S1, S2, S3, S4 and S5 was 38.92%, 56.59%, 42.56%, 39.11% and 41.81%, respectively. *Acidobacteria* was another dominant phylum in all of the samples, except in S1 the abundance of *Actinobacteria* was higher than that of *Acidobacteria. Firmicutes* was the predominant phylum at 40.84% in S1, but the abundance of *Firmicutes* in the other samples was very low.

**Figure 4:**
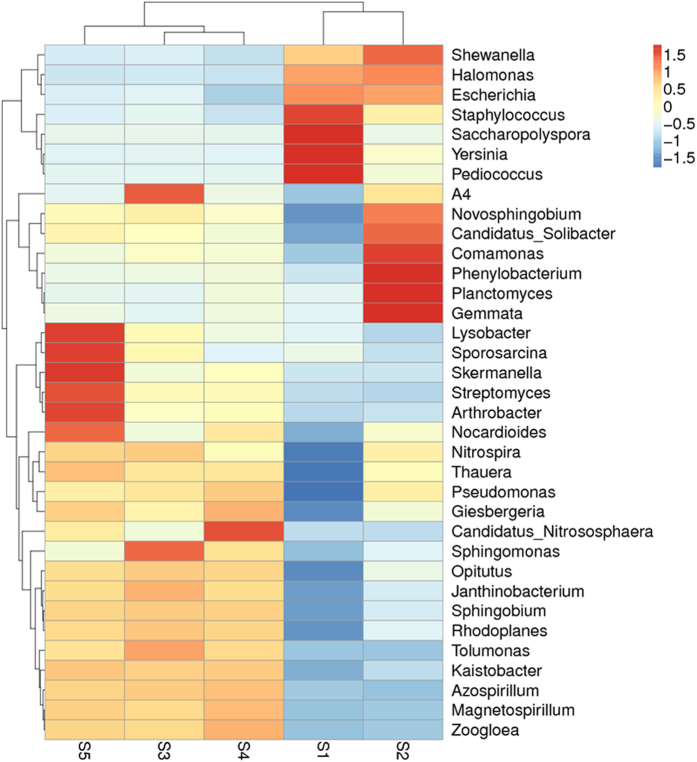
Heat maps of species abundance clustering. The genus classification position clustering (horizontal) and top 35 genera sample clustering (vertical clustering). Different color means the different relative abundance of the genus in the all five samples (red means great abundance).

A further analysis was conducted to discover the relative bacterial community abundance at the genus level, and the results are shown in Fig. 4. The relative abundance of species is indicated by a Z value in each row of the heat map after standardization. A total of 35 genera were identified among the five samples and all these genera were at a maximum in S2, followed by S1, S4, S3 and S5. *Halomonas* was the most abundant genus observed in all five samples, while *Lysobacter* was the least. The genera distribution in the five samples fell into six groups. Group 1 (*Staphylococcus, Saccharopolyspora, Yersinia* and *Pediococcus)* contained the dominant genera with the highest abundance in S1. Group 2 (*Comamonas, Phenylobacterium, Planctomyces* and *Gemmata)* was predominant in S2. Group 3 (*Lysobacter. Sporosarcina, Skermanella, Streptomyces* and *Arthrobacter*) was predominant in S5. However, some genera were predominantly distributed in more than one sample. Group 4 (*Shewanella, Halomonas* and *Escherichia*) was predominant (2.15%, 13.75%, 1.99% and 3.10%,15.07%, 1.86%, respectively) in S1 and S2. Group 5 (*A4, Candidatus_Nitrososphaera* and *Sphingomonas*) was predominant in S3 and S4, and group 6 (*Nitrospira, Thauera* and some other genera) was predominant in S3, S4 and S5. Each group had a different ecological function. The genera in group 1 were more adapted to a no-till soil environment, while the group 2 genera could survive in a corn field with high tolerance to HM stress, and group 3 had no HM stress tolerance. Importantly, group 4 genera were absolutely highly tolerant to HM stress in both no-till fields and corn fields. The genera in group 5 could only survive in a corn field with medium tolerance to HM stress. The group 6 genera were more adapted to a cornfield environment. The relative abundances of the ten dominant phyla found in this study were consistent with the findings of Liu, *et al*.^58^ for agricultural soils in China, planted with soybeans, maize, or wheat, and Burns, *et al*.^59^ for vineyard soil in the USA.

**Figure 5:**
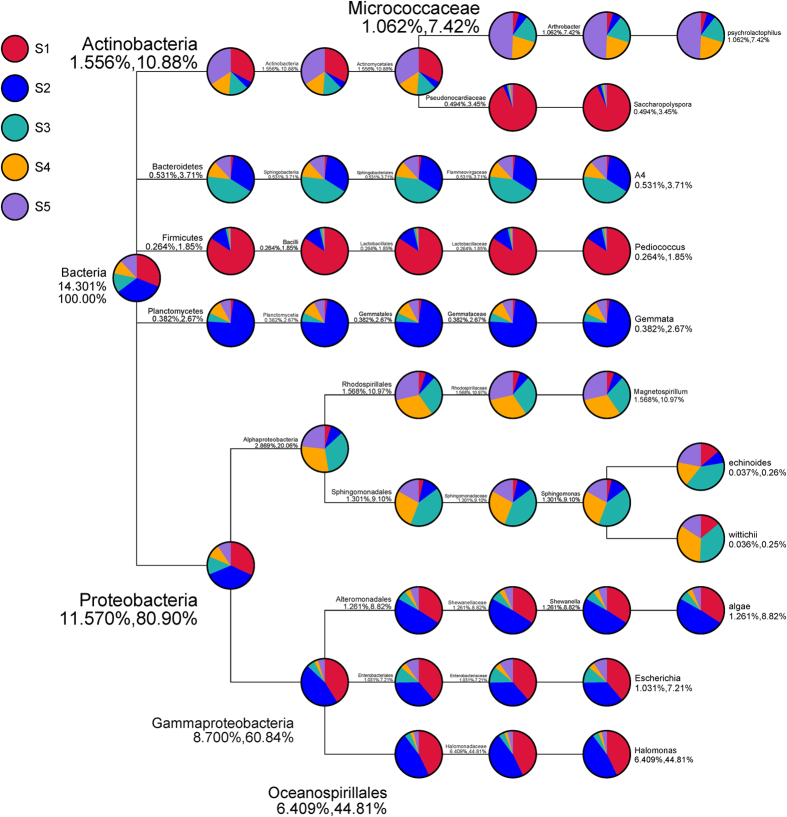
The relative abundance of each class based on 16S rDNA sequence analysis. The relative abundance is expressed in percentage and classification tree of complex samples. different color of circle fan means different sample; the size of the fan means the relative abundance of proportional size on classification level of samples; The numbers below the classification name stands for the average percentage of relative abundance on this classification level in all samples. There were two numbers, the former one means the percentage of all species, the latter one means the percentage of selected species.

**Figure 6:**
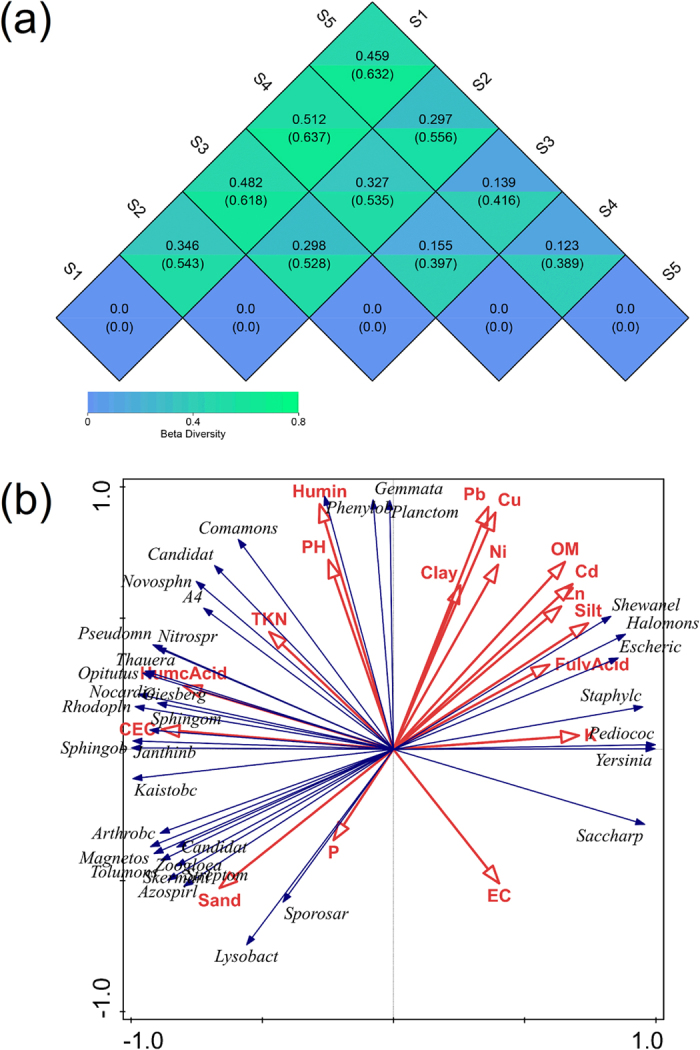
Beta-diversity heat map and Redundancy analysis. (**a**) Relationship between heat map of β-diversity index for all five samples soil bacteria with increasing the sampling distance from zinc smelter. β-diversity value is the discrepancy coefficient between the two samples. Located the number of reads belong to the same species in different samples into the same table, the profiling table was generated. Here weighted unifra (upper) and unweighted unifra distance were set as the measure index. (**b**) Redundancy analysis (RDA) of environment factors and top 35 genera of soil samples. Plots could interpreted quantitatively, using the length of the arrow to indicate how much variance was explained by that factor. The direction of the arrow for individual environmental factor indicates an increasing concentration of that factor. The RDA ordination plot was constructed by projecting an object (site) at a right angle on a response (dominant genera) or an explanatory (soil) variable, which in turn approximates the value of the object along that variable. The angles between the response and explanatory variables or between response variables themselves reflect their correlations.

In addition, the beta-diversity was also analyzed to determine the broad trends of similarities and differences among the samples via a cluster analysis for the five different samples. The results are shown in Fig. 5a. The correlation coefficient among all five samples demonstrated that the HM concentration was directly correlated with distance from the zinc smelter and the important parameter of bacterial beta-diversity shown in Fig. 6a. The value smaller, the difference in species diversity smaller, with the weighted unifra (upper) and un-weighted unifra distance set as the measure index. An analysis of the quantitative data with increasing distance, indicated a higher beta-diversity between S1 and S5, while the lowest was found between S4 and S5. The bacterial beta-diversity was relatively high between S1 and S5, and the difference of the total bacterial population was also huge, possibly because the HM content was toxic, as indicated in (Fig. 2a,b).

The phylum-level bacterial communities and beta-diversity both indicated that *Proteobacteria, Acidobacteria, Actinobacteria* and *Firmicutes* were most greatly affected by the distance from the smelter and the HM concentration (Figs 2a and 3b). The magnitude of compositional turnover along the HM concentration and humus content gradient was greatest for *Proteobacteria, Acidobacteria, Actinobacteria* and *Firmicutes* followed by *Gemmatimonadetes, Bacteroidetes, Planctomycetes, Verrucomicrobia* and others (maximum sampling distance of point of 10 km from smelter (Fig. 4). A higher maximum diversity for the *Acidobacteria* (11.88%) was observed than for the *Actinobacteria* (6.90%), while highest for the *Proteobacteria* (56.59%) was in S2. The bacterial beta-diversity was highest between the nearest sampling point (S1) and the furthest sampling point (S5), where the difference of HM concentration was very big, which indicate the bacterial beta-diversity showed a strong positive correlation with the HM concentration^60^. Therefore, a significant difference was observed for these phyla from the other sampling sites as a function of distance (Figs 4 and 5a).

*Proteobacteria* and *Actinobacteria* constantly showed maximum abundance among all five sample sites (Fig. 3b), including at the furthest sampling point (S5). However, four other soil properties (i.e., total organic matter, and the humin, HA and FA content) also played an important role in the maximum compositional turnover for *Proteobacteria* and *Actinobacteria* (Fig. 2c). *Actinobacteria* and *Acidobacteria* exhibited twice as much turnover with sampling distance between S2 and the other sampling points (compare the relative abundance at the phylum level in Fig. 3b). Furthermore, at the genus level (Fig. 4), the maximum relative abundance of the genera *Shewanella* and *Halmonas* was observed in S2, while the lowest were *Zoogloea* and *Magnetospirillum*. However, the relative bacterial diversity decreased with sampling distance and lowest bacterial population was observed in S5. Heat maps of genus abundance showed clusters of relative bacterial diversity and the shapes of the I-splines for *Shewanella, Halmonas* and *Escherchia* were similar to those for genera within *Proteobacteria* (Fig. 4). However, the amount of deviation explained for these genera in S2 was lower than in the other samples 48.4, 31.6 and 13.6%, respectively.

Overall, our results indicated that the sampling distance, the HM content and the bacterial β-diversity have direct correlation with soil humus carbon content, HA and FA, which is an important driver of soil bacterial community composition variation in all five soil samples. However, the effect of the soil humus carbon content, HA and FA on the compositional shifts is not consistent between OTUs and Taxa, but it varies between phylum and genus as a function of the position along the sampling points. Furthermore, the sampling distance and the HM content are major factors and had a strong effect on several common bacterial phyla and genera. Finally, our findings clearly indicated that sampling distance may indirectly influence the HM content in the area surrounding the zinc smelter, and as a result, the soil bacterial community was affected.

### Co-relationships between bacterial communities and soil properties

In this study we explored the relationships between the bacterial community and soil properties in terms of the total HM and available HM (Cd and Zn) content as well as the contents of soil organic fractions such as HC, HA and FA on the bacterial community composition in soil surrounding a zinc smelter. A Redundancy Analysis (RDA) was also performed to examine the physicochemical factors affecting the top 35 genera, as ranked by the relative abundance of all bacteria (Fig. 6b). The plots could be interpreted quantitatively, using the length of the arrow to indicate how much of the variance was explained by a given factor. The direction of the arrow for a particular environmental factor indicates an increasing concentration of that factor. The RDA ordination plot was constructed by projecting an object (site) at a right angle on a response (dominant genera) or an explanatory (soil) variable, which in turn approximates the value of the object along that variable. The angles between the response and explanatory variables or between the response variables themselves reflect their correlations^61^. Both the average Cd and Zn concentrations were significantly correlated with the SOM content. The three genera that were highly resistant to HM stress as indicated in Fig. 4 were significantly correlated to the Cd and Zn concentration. However, no remarkable positive correlations were apparent for either genera or soils physicochemical properties. Nevertheless, the soil CEC and SOM showed a significant correlation with soil HM and the bacterial community.

Our results also showed that high concentrations of metals (e.g., Cd, Cu, Ni, Pb and Zn) in the soil decreased the total population of bacteria, but the tillage conditions increased the Taxon Tags and the Unique Tags, as evidenced by the observation of the maximum total HM content in S2 (corn field soil), which was located closest to the main chimney of zinc smelter similarly to S1, but S1 was uncultivated soil. From Fig. 2, we inferred that HA and FA were two other representative factors that affected the bacterial diversity but the content of other HM such as Cu and Ni did not. Several previous studies reported that the relative abundance of *Proteobacteria* decreased while the relative abundance of *Actinobacteria* increased with an increasing HM concentration as evidenced by 16S rDNA-based banding patterns^62^, and HA and FA played crucial role in the bioavailability of soil HM and consequently affected the bacterial diversity^63^. In addition, the bacterial population at highly polluted sites (S2, S1 and S3) were much more different than that at lightly polluted sites (S4 and S5) because the communities at S4 and S5 were more similar to each other than the others, as shown in Figs 3b, 4 and 5. The bacterial communities at S3 and S4 were less similar than at the other sampling points. Insam^64^ observed that the soil microbial biomass reflected the size of total microbial community in the soil and Chen^65^ also reported that the soil microbial biomass varied greatly, ranging from 95.1 μg C g^−1^ near a Zn smelter to 497 μg C g^−1^, 10 km away from the smelter. The sequencing depth and number of phyla in each samples defined by 97% of the sequences showed similarity with known genera, but increasing the sequencing depth suggested that the total bacterial population in each HM contaminated soil sample had not been completely determined^21^,^58^,^66^,^67^.

Similarly, most taxa (e.g., *Proteobacteria*) were closely associated with the HM concentration in one or more of the soil fractions (*P* < 0.05), showing a lower relative abundance with an increased concentration of HM and distance. However, *Actinobacteria* and *Acidobacteria* were two exceptions with a higher relative abundance with an increased HM concentration and distance. A comparison of our study with a previous study by Xu^21^ on the soil microbial diversity of the area surrounding the zinc mines of Feng County suggested that the composition of the *Actinobacteria* was more copiotrophic, while the *Proteobacteria* was more oligotrophic, although both are diverse bacterial groups that are characterized by a wide variety of life history strategies^68^.

Figure 6a shows that the correlation between HM and the physicochemical properties of all soil samples was not statistically significant. Nevertheless, our results indicate that more highly available HM in the soil might have a greater adverse effect on the soil bacterial biomass. To screen the dominant factors of soil chemical parameters affecting soil bacterial community, a stepwise regression analysis was performed for the soil HA/FA ratio, the HM availability, soil pH, and humin carbon content. Figure 6 shows that humic substances typically represent a large portion of the natural organic matter (NOM) in the soil, which is an excellent nutrient for bacterial growth. Similarly, our result indicates that the HM and humic substances concentrations are positively correlated with the bacterial community (*r* = 0.950, *P* = 0.012), which may be because a higher concentration of HA/FA decreases the bioavailability of HM and thus increases the bacterial community with an increasing of distance. However, bacterial diversity was negatively correlated with the other physicochemical parameters, but a significant correlation was observed between the bacterial relative abundance and the beta-diversity (r = −0.501, *P* = 0.139).

## Conclusions

In summary, to our knowledge, this study is the first report about uncultivable bacterial communities associated with contaminated soil in a zinc smelting area. The results showed that the bacterial communities were dominated by the phyla *Proteobacteria, Acidobacteria* and *Actinobacteria* in cornfield soils, except *Fimicutes* phylum was dominated in hilly area soil, all surrounding the zinc smelter. The HM content of agricultural top soil was inversely proportional to the distance from the smelter. The soil CEC, HA/FA and microbial OTUs increased with the sampling distance increased. Therefore, smelter dust emissions were generally the major source of HM contamination in the nearby soil, and long-term atmospheric deposition of metal-containing particles in a smelting area can cause extensive damage to the ecosystem. *Shewanella, Halomonas* and *Escherichia* genera were highly tolerant to HM stress in both cultivated and non-cultivated soils. We observed that an increase in the soil HM concentration decreased the bacterial diversity, and the soil organic fraction (SOM, HC, HA and FA) contents significantly affected the beta-diversity and relative abundance in all soil samples. Further studies are required to investigate the tolerance levels to HM for the genera identified in the five samples because of their potential utility in metal migration and phytoremediation of HM-contaminated sites.

## Additional Information

**How to cite this article**: Shen, F. *et al*. Atmospheric Deposition-Carried Zn and Cd from a Zinc Smelter and Their Effects on Soil Microflora as Revealed by 16S rDNA. *Sci. Rep.* **6**, 39148; doi: 10.1038/srep39148 (2016).

**Publisher's note:** Springer Nature remains neutral with regard to jurisdictional claims in published maps and institutional affiliations.

## Figures and Tables

**Table 1:** Physicochemical properties of soils collected from Feng County zinc smelter area.

Sample No.	Soil texture	pH	EC (mS·cm^−1^)	CEC (cmol·kg^−1^)	SOM (g·kg^−1^)	TKN (g·kg^−1^)	TP (g·kg^−1^)	TK (g·kg^−1^)
S1	Silt	8.45^*b*^ (0.05)	157.6^*a*^ (0.75)	16.3^*d*^ (0.09)	17.0^*c*^ (0.5)	0.215^*b*^ (0.009)	0.565^*b*^ (0.002)	6.03^*a*^ (0.130)
S2	Silt	8.68^*a*^ (0.10)	146.0^*c*^ (0.45)	25.5^*c*^ (0.09)	24.8^*a*^ (0.5)	0.387^*a*^ (0.038)	0.575^*b*^ (0.039)	5.38^*ab*^ (0.215)
S3	Silt	8.52^*ab*^ (0.00)	152.6^*b*^ (0.75)	25.9^*c*^ (0.08)	20.0^*b*^ (0.7)	0.411^*a*^ (0.005)	0.703^*a*^ (0.003)	5.64^*a*^ (0.352)
S4	Silt loam	8.48^*ab*^ (0.08)	139.7^*d*^(0.80)	28.3^*b*^ (0.18)	20.2^*b*^ (0.9)	0.239^*b*^ (0.016)	0.576^*b*^ (0.001)	4.94^*b*^ (0.351)
S5	Silt loam	8.63^*ab*^ (0.03)	156.5^*a*^(1.85)	34.3^*a*^ (0.16)	16.3^*c*^ (0.6)	0.370^*a*^ (0.020)	0.607^*b*^ (0.037)	4.16^*c*^ (0.186)

The mean value replicate sample is shown, with the standard error in parentheses. Plots that were not significantly different with respect to metal concentration (*P *> 0.05, one-way analysis of variation with LSD test) are marked with the same level in superscript. EC (electrical conductivity), CEC (cation exchange capacity), SOM (Soil organic matter), TKN (Total Kjeldahl nitrogen), TP (Total phosphorus), TK (Total potassium).
